# Supplementary material for: Deletion of wfs1 Impairs Oligodendrocyte Precursor Cells Dorsal Distribution and Myelination Through the wfs1-hmgcs1 Axis in Zebrafish
Source: Biology (Basel). 2026 Aug 16;15(16):1402. doi: 10.3390/biology15161402 (PMC13510151; doi:10.3390/biology15161402)
Supplement: Supplementary file 1 [file biology-15-01402-s001.zip › File S1-Transcriptome sequencing data and differentially expressed genes (DEGs) in wfs1-- zebrafish at 5 dpf/2.KEGG/wfs1KOvsWT/UP/src/dre00565.html]

dre00565


- dre:560225

- Up regulated genes

560225(2.51169116131343)

- dre:560225

- Up regulated genes

560225(2.51169116131343)

- dre:560225

- Up regulated genes

560225(2.51169116131343)

- dre:567170

- Up regulated genes

567170\_1(4.28009552437754)

- dre:406370

- Up regulated genes

406370(7.33922178722892)
- dre:560649

- Up regulated genes

560649(1.22362655394035)

- dre:406370

- Up regulated genes

406370(7.33922178722892)
- dre:560649

- Up regulated genes

560649(1.22362655394035)

- dre:492735

- Up regulated genes

492735(1.97226761409193)

- dre:560225

- Up regulated genes

560225(2.51169116131343)

- dre:565743

- Up regulated genes

565743\_1(2.40489825782141)

Close
